# Improving Type 2 Diabetes Care with Extended-Release Metformin: Real-Life Insights from a Physician Educational Program

**DOI:** 10.2174/0118715303294909240221102552

**Published:** 2024-02-28

**Authors:** Laura Molteni, Giuseppe Marelli, Giona Castagna, Luciano Brambilla, Maurizio Acerbis, Fabio Alberghina, Antonio Carpani, Erika Chiavenna, Maria Grazia Ferlini, Carmen Impellizzeri, Roberto Paredi, Alberto Rigamonti, Giuseppe Rivolta, Olga Eugenia Disoteo

**Affiliations:** 1 Centre for Diabetology, Endocrinology and Treatment of Metabolic Diseases, Sacra Famiglia Hospital, Erba, Italy;; 2 University of Milano Bicocca, Milan, Italy;; 3 General Practitioner, ATS Insubria, Erba District, Como, Italy;; 4 Division of Endocrinology and Diabetology, Sant’Anna Hospital - ASST Lariana, Como, Italy

**Keywords:** Type 2 diabetes mellitus, extended-release metformin, satisfaction, adherence, tolerability, glycated hemoglobin, side effects

## Abstract

**Background:**

Compared to Immediate-Release (IR) metformin, Extended-Release (ER) metformin reduces side effects and pill burden while improving adherence; however, there is little real-life data on patient satisfaction with this innovative formulation to guide physicians toward a more holistic approach.

**Objective:**

Our goal is to train general practitioners on holistic patient management, with the aim of increasing patient satisfaction and treatment adherence, reducing side effects, and improving quality of life in patients with poor tolerance to metformin-IR.

**Materials and Methods:**

We designed an educational program for physicians called SlowDiab, aimed at establishing a holistic patient approach. In this context, adult patients with T2DM who experienced gastrointestinal discomfort with metformin-IR were enrolled and switched to metformin-ER. Data on glycemic control were collected at baseline and 2 months after switching. A survey was carried out on patients to assess their level of satisfaction.

**Results:**

In 69 enrolled patients (mean (min-max) age, 68.2 (41-90)), side effects decreased after switching from 61.8% to 16.2% (*p* < 0.01), and the mean perceived burden of adverse events on a scale of 1 to 10 also decreased (6.17 *vs*. 3.82; *p* < 0.05). Among patients previously intolerant to metformin-IR, 74.3% reported no longer experiencing any side effects after the switch. The mean number of tablets taken daily (2.28 *vs*. 1.66; *p* < 0.01) and mean plasma glycated hemoglobin (HbA1c) values (7.0% *vs*. 6.7%; *p* < 0.05) decreased, while 93.8% of patients were satisfied with the treatment change. Moreover, 84.2% reported an improvement in glycemic control after the switch.

**Conclusion:**

In a real-life setting, an educational program for general practitioners confirmed that metformin ER reduces side effects and improves pill burden, therapeutic adherence, and patient satisfaction compared to metformin IR.

## 1 INTRODUCTION

Diabetes is a chronic, widespread disease that causes disabling complications for patients and high costs for healthcare systems [1]. Globally, it affects more than 500 million adults, and the number of patients with type 2 diabetes mellitus (T2DM) has increased dramatically over the past 30 years worldwide [1-3]. Metformin, a molecule that has revolutionized the treatment of T2DM, is included in the World Health Organization’s list of essential drugs and is recommended as a first-line therapy by all national and international guidelines [4-7]. Its powerful hypoglycemic effect, safety, wide availability, and low cost have made it the most frequently prescribed antidiabetic drug in the world [8]. Investigations into its mechanism of action, which has not yet been fully elucidated, suggest that reduced hepatic glucose production through inhibition of gluconeogenesis is mainly responsible for its euglycemic efficacy [9-18]. Furthermore, it also increases peripheral glucose uptake, particularly by muscle [12-22]. However, additional mechanisms remain a subject of study, including the downregulation of lipogenic enzymes and modulation of cellular respiration [22-24].

Metformin has an oral bioavailability of approximately 50%; the remainder accumulates in the intestinal mucosa at concentrations that are 30 to 300 times higher than those in plasma [12]. The effects of metformin in the intestine are still unclear, but it has been hypothesized that it may delay intestinal glucose absorption and increase glucagon-like peptide 1 (GLP1) secretion *via* sodium-glucose transporter 1 (SGLT1). Moreover, it may also influence the intestinal microbiome by favoring short-chain fatty acid-producing bacteria and modulating inflammation [8, 12].

An increasing body of evidence suggests that this molecule may have non-glycemic effects that could be useful for treating oncological, neurodegenerative, and rheumatological diseases [8, 9, 13, 14, 25-27].

Metformin is widely recognized as a safe treatment for T2DM, given its low risk of hypoglycemia and neutral effect on body weight. Its hypoglycemic effect results in an estimated reduction in glycated hemoglobin (HbA1c) of between 1 and 2% [15, 16]. However, about 30% of patients experience gastrointestinal side effects with the immediate-release (IR) formulation, and 5-10% cannot tolerate it at all [10, 17]. Intolerance usually occurs at the start of treatment, with symptoms, such as diarrhea, nausea, lack of appetite, heartburn, and vomiting, representing a significant obstacle to treatment continuation [9, 10, 17]. Metformin is poorly absorbed in the stomach, while most of the drug is absorbed in the proximal small intestine [9]. Intestinal accumulation of the active ingredient may be one cause of the reported gastrointestinal side effects [12, 18].

Despite its proven efficacy, tolerability issues prevent some patients from taking adequate doses, often resulting in poor adherence, worsening health outcomes, and higher healthcare costs. Extended-release (ER) metformin was developed as a better-tolerated formulation that would improve compliance and glycemic control. Metformin-ER provides a slow release of the drug in the upper gastrointestinal tract and maximize absorption [14, 19]. In particular, the Gel Shield Diffusion System^®^ consists of a polymer matrix that gradually releases the active ingredient, allowing metformin to dissolve when exposed to gastrointestinal fluids [10, 19]. This slower absorption is associated with a lower incidence of gastrointestinal side effects compared to metformin-IR [10, 28-31]. It can also simplify the treatment regimen, compared to the original formulation, by allowing once-daily administration [10, 29].

Although metformin-ER has been available in clinical practice since 2000, little real-world evidence exists on its use and patient satisfaction [23, 28].

Moreover, despite the well-established evidence supporting the efficacy of metformin-ER, its utilization is often suboptimal due to therapeutic inertia or a lack of investigation by physicians into potential side effects associated with metformin-IR [29-31].

Recognizing these challenges, we developed the SlowDiab educational initiative, with the primary objective of addressing the underutilization of metformin-ER by physicians while emphasizing the importance of a holistic patient management approach.

SlowDiab aims to investigate whether transitioning from metformin-IR to metformin-ER can alleviate the gastrointestinal side effects reported by patients in a real-world context. We hypothesized that this switch would improve treatment adherence, glycemic control, and the quality of life for individuals with diabetes.

The genesis of SlowDiab stems from the acknowledgment that therapeutic inertia and a lack of awareness about the potential adverse effects of metformin-IR may hinder optimal patient care. Through this educational endeavor, we seek to bridge the gap between existing knowledge and clinical practice, fostering a more informed and proactive approach to diabetes management among healthcare professionals.

In addition, SlowDiab aims to evaluate whether this holistic and personalized approach to patient care translates into increased patient satisfaction and improved glycemic control. By addressing therapeutic inertia and enhancing awareness, we aspire to contribute to a positive shift in patient outcomes and well-being within the context of diabetes management.

## 2 MATERIALS AND METHODS

Before enrolling patients, participating general practitioners underwent a dedicated training program focused on the critical role of treatment adherence. Emphasis was placed on the positive correlation between patient adherence and the use of therapies with minimal side effects and a reduced daily pill burden.

In the context of this physician education program, conducted between October, 2021 and May, 2022 in the Province of Como, Italy, general practitioners enrolled adults aged ≥ 18 years with T2DM who were managed in diabetes center outpatient clinics or in general practice. Enrolled patients had experienced gastrointestinal discomfort with metformin-IR and were switched to metformin-ER. The decision to switch from metformin-IR to the extended-release (ER) formulation was made by the attending physician in response to observed side effects, aiming to mitigate the impact on patient well-being and enhance treatment adherence.

During the index visit, each patient received a survey (Appendix) consisting of 13 items that assessed any side effects experienced with each of the metformin formulations to be completed at home and returned at the next visit after approximately 8 weeks of metformin-ER therapy. In addition, demographic and clinical data were collected, including patient age, sex, disease duration, dates of visits, reasons for switching to the ER formulation, dosages of the two metformin formulations, and HbA1c blood values at therapy switch and after 8 weeks of metformin-ER treatment.

Data collected during the trial were stored in a database, and statistical analyses were conducted using SPSS (Statistical Package for the Social Sciences).

The chi-square test was used to determine whether the observed frequencies corresponded to the expected frequencies. The one-sample t-test was used to assess the significance of changes in side effects before and after the switch to metformin-ER. The paired-sample t-test was used to assess the change in HbA1c values and mean number of tablets before and after the switch.

## 3 RESULTS

A total of 69 patients required switching from metformin-IR to metformin-ER. The patient questionnaire was completed by 68 subjects; 39 men and 30 women, with a mean age of 68.2 years and a mean T2DM disease duration of 8.2 years.

The physician’s most frequent motivation for switching to metformin-ER (multiple choice response) was intolerance of metformin-IR (51.5%), followed by a need for simplification of the dosage schedule (38.2%) and the innovativeness of the ER formulation (22.1%). After the switch to metformin-ER, total daily metformin dosage was reduced (Table **1**).

After 8 weeks of follow-up from the therapeutic switch, there was a significant reduction with metformin-ER in the mean daily pill burden, accompanied by significant improvements in mean plasma HbA1c levels, percentage of subjects reporting side effects, and mean perceived invasiveness of side effects (Table **2**).

**Fig. (1):**
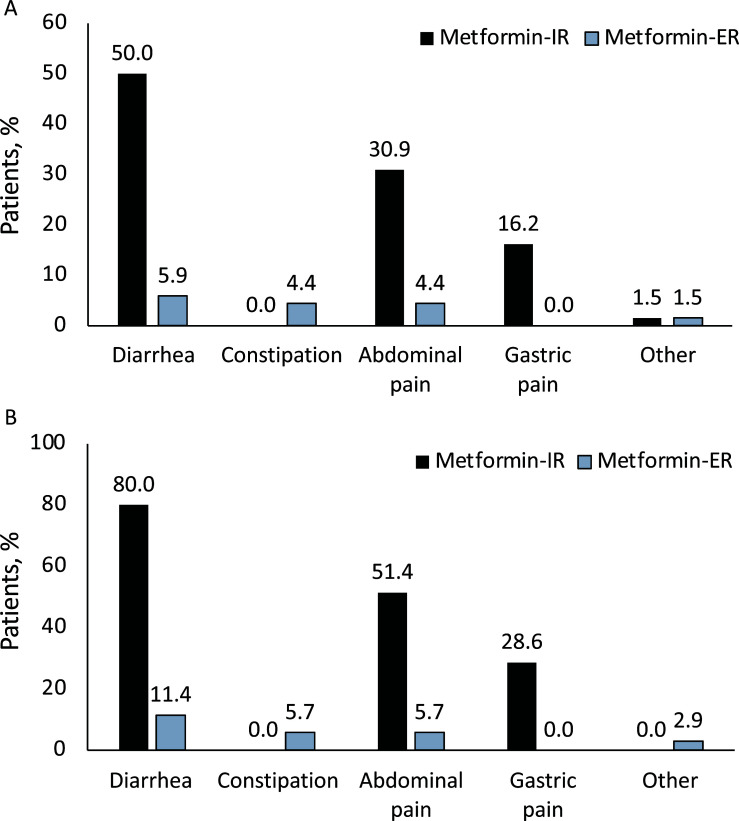
**A**. Frequency of side effects reported by patients before and after the therapeutic switch in the general population (n = 68); **B**. Frequency of side effects reported by patients before and after the therapeutic switch in the subgroup of those who changed the formulation due to intolerance to metformin-IR (n = 35).

In the subgroup of patients whose motivation for switching therapy was complete intolerance to metformin-IR (n = 35), a substantial reduction in side effects was reported after 8 weeks of treatment with metformin-ER. All patients in this category reported ≥ 1 side effect with metformin-IR, whereas after the switch to metformin-ER, this percentage was reduced to 25.7%. In the overall study population, the frequency of patients experiencing diarrhea or abdominal pain after 8 weeks of treatment with metformin-ER was reduced by 88% and 86%, respectively, compared to the pre-switch period. Gastric pain, present in 16.2% of patients on metformin-IR, was not reported during metformin-ER treatment, whereas one gastrointestinal event (constipation) occurred during metformin-ER treatment (Fig. **1A**).

In the subgroup of subjects who were intolerant to metformin-IR, the efficacy of metformin-ER in reducing gastrointestinal side effects was also confirmed. The number of patients with diarrhea decreased by 86%, those with abdominal pain by 88%, and gastric pain was not reported (Fig. **1B**).

**Fig. (2):**
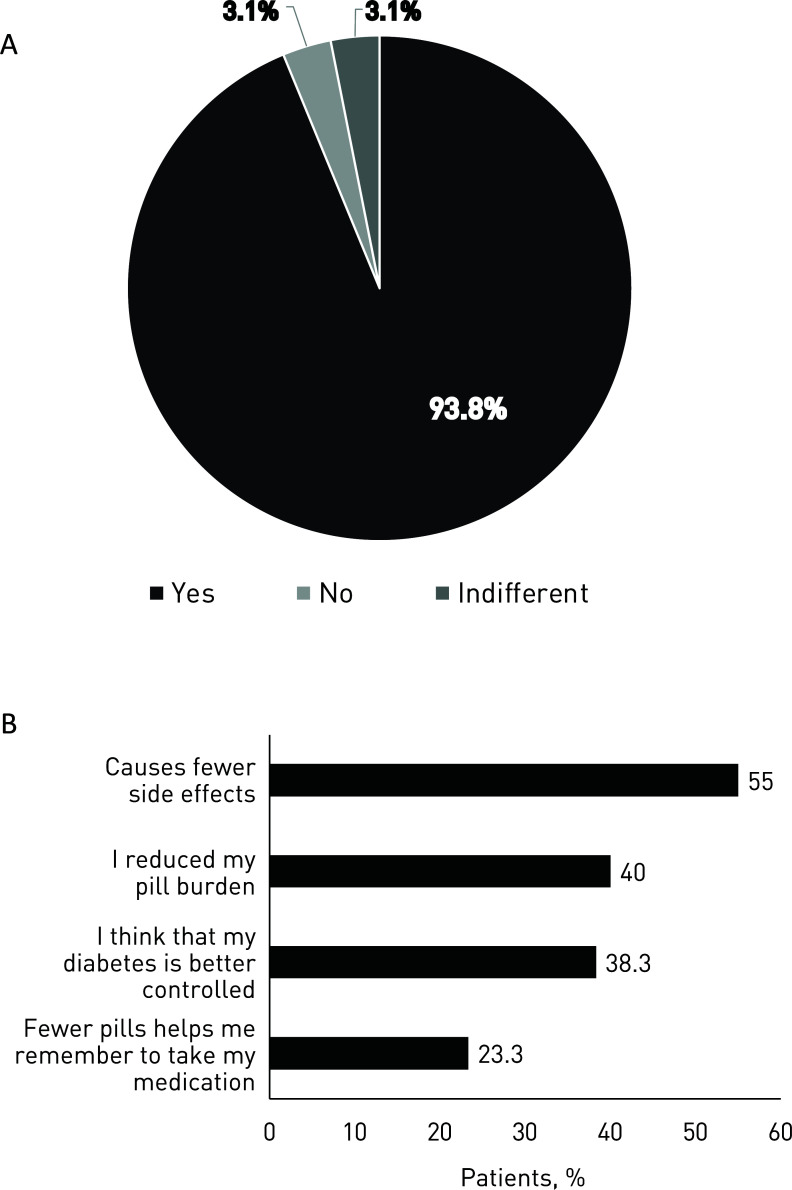
**A**. Satisfaction with the switch to metformin-ER formulation; **B**. Reasons for satisfaction with metformin-ER treatment.

The patient questionnaire also assessed patient satisfaction with the extended-release formulation. After the switch to metformin-ER, 65.2% of patients reported a reduction in the number of tablets; of these, 92.7% of patients stated that the reduction was “comfortable/important for their daily routine”, while 7.3% of the patients were indifferent. During 8 weeks of treatment with metformin-ER, 84.2% of patients stated that they perceived an improvement in T2DM control, and 93.8% were satisfied with this therapeutic change (Fig. **2A**); the most frequent reasons for this satisfaction (multiple choice response) were the decrease in side effects and the reduction in the number of tablets (Fig. **2B**).

Finally, 95.3% of patients stated that they would recommend switching to metformin-ER to other patients.

## 4 DISCUSSION

The hypoglycemic properties of metformin are well established, and its use in recent decades has gradually established it as one of the most prescribed drugs [32, 33]; however, poor gastrointestinal tolerability with metformin-IR is widely reported [34-40], resulting in gastrointestinal adverse events in approximately 30% of diabetic patients. These effects may cause discomfort and, in some patients, may be an obstacle to achieving the therapeutic target [41-44].

The primary objective of our physician-focused educational initiative, SlowDiab, was to address therapeutic inertia and enhance awareness among healthcare professionals regarding the potential benefits of metformin-ER for improving patient outcomes. Our study aimed to assess subjective patient satisfaction with metformin-ER treatment in a real-world setting.

The results confirmed that metformin-ER possesses a substantially better tolerability profile than its immediate-release counterpart in a real-world setting. This holds true for both the general T2DM population and the specific subset of T2DM patients who had been intolerant to metformin IR. In the latter category, our study revealed that metformin-ER successfully eliminated side effects in three out of four intolerant patients, making it a viable alternative for those who had encountered challenges with the immediate-release formulation.

In addition, we observed that a large percentage of patients were satisfied with the new treatment (93.8%), not only because they experienced fewer side effects with metformin-ER but also because the daily tablet burden was reduced. Patients were satisfied because this facilitated their daily routine (40% because they reduced the number of tablets, and 23.3% because fewer tablets helped them to remember to take their medication); meanwhile, 38.3% cited perceived subjective improvement in disease control as a reason for satisfaction.

We also verified a significant improvement in glycemic control after the therapeutic switch, with an average reduction of about 3.8 mmol/mol HbA1c, despite no increase in metformin dosage. One hypothesis is that the reported increase in patient satisfaction may have led to greater adherence to therapy, resulting in improved glycemic control.

This real-world evidence not only aligns with the educational program's goal of optimizing patient satisfaction and overall well-being but also emphasizes the potential impact of physician awareness on treatment choices. The holistic and personalized approach to patient care in SlowDiab revealed that certain medications, such as metformin-ER, can significantly enhance patient quality of life and treatment adherence. By confirming the real-world data on tolerability and satisfaction, our study underscores the importance of integrating such knowledge into clinical practice, ultimately contributing to improved outcomes in the management of T2DM.

Given the chronic nature of T2DM, adherence to treatment is crucial for preventing complications associated with the disease. Poor adherence in diabetes is often due to the complexity of the dosage regimen, polypharmacy (especially in older adults), side effects, and the need to use injectable drugs [40-46]. Many studies have shown that adherence to metformin-IR is often sub-optimal [47-51], not only because of the known gastrointestinal side effects but also because of multiple daily dosing [52-56]. In this program, simplification of the metformin dosing regimen was the second most common reason given by physicians for switching formulations. The extended-release formulation can be administered once daily, which is an advantage in terms of better compliance [57-61].

Despite its proven efficacy for T2DM, favorable tolerability profile, and the possibility of simplifying the dosing schedule, metformin-ER is still not widely used in Italy. Recent data indicate that the market share of the IR formulation is 15%, despite evidence that metformin-IR intolerance occurs in up to one-third of the T2DM population. This low use of the ER formulation may be attributed to reluctance on the part of patients treated with metformin-IR to report side effects correctly and completely [62-67]. Furthermore, therapeutic inertia on the part of physicians has been documented recently in the Annals of the Italian Diabetes Medical Association [45]. Moreover, evidence shows that therapeutic inertia in diabetic pathology is associated with worse micro- and macro-vascular outcomes [62, 63, 68-72].

## CONCLUSION

In conclusion, the SlowDiab educational initiative aimed to address therapeutic inertia and increase awareness among physicians about the benefits of metformin-ER. It also assessed patient satisfaction with metformin-ER in real-world conditions.

The findings confirmed better tolerability of metformin-ER compared to the immediate-release formulation, providing a viable alternative that simplifies therapy and improves quality of life. Additionally, our study revealed a notable patient satisfaction with metformin ER that was correlated with improved glycemic control. We propose that this enhancement may be linked to an increase in treatment adherence.

The results of this educational program demonstrated that even modest therapeutic changes can significantly enhance various aspects of life for patients with Type 2 Diabetes Mellitus (T2DM). These improvements extend beyond clinical values, encompassing enhanced quality of life and increased patient satisfaction. Importantly, this positive shift may also contribute to heightened satisfaction among clinicians, providing an increasingly holistic view within the medical profession.

## Figures and Tables

**Table 1:** The proportion of patients treated with the indicated daily metformin doses before and after the therapeutic switch.

**Daily Metformin Dosage**	**Metformin-IR** **(% Patients)**	**Metformin-ER** **(% Patients)**
< 1000 mg	38.2	33.8
1000 mg – 1500 mg	26.5	41.2
1500 mg – 2000 mg	27.9	25.0
> 2000 mg	7.4	--

**Abbreviations:** IR, immediate-release; ER, extended-release

**Table 2:** Clinical parameters before and after the therapeutic switch.

**Parameter**	**Met-IR**	**Met-ER**	***t***	***p***
Mean tablets per day/patient, n (SD)	2.28 (± 0.484)	1.66 (± 0.507)	8.88	.000
Mean plasma HbA1c, % (SD)	7.0 (± 0.588)	6.7 (± 0.395)	8.99	.000
Patients reporting ≥1 side effect, % (n)	61.8 (42)	16.2 (11)		.004
Perceived side effect invasiveness on a 1 to 10 scale (SD)	6.17 (± 2.408)	3.82 (± 2.228)	3.00	.013

**Abbreviations:** SD, standard deviation; HbA1c, glycated hemoglobin A1c; Met. metformin.

## Data Availability

The data that support the findings of this study are available from the corresponding author, LM, on special request.

## LIST OF ABBREVIATIONS

ER: Extended-Release
GLP1: Glucagon-Like Peptide 1
IR: Immediate-Release
T2DM: Type 2 Diabetes Mellitus

## APPENDIX

### QUESTIONS ON PREVIOUS TREATMENT (METFORMIN-IR)

Any side effects with the previous treatment (metformin-IR)?

▪ Yes

▪ No

If yes, specify:

▪ Diarrhea

▪ Abdominal pain

▪ Gastric pain

▪ Constipation

▪ Other, specify ____________

If yes, specify the invasiveness of side effects:

(1 = few effects, 10 = many side effects)

1 2 3 4 5 6 7 8 9 10

### QUESTIONS ON NEW TREATMENT (Metformin-ER)

Any side effects from the previous treatment (metformin-IR) to the new one (metformin-ER)?

▪ Yes

▪ No

If yes, specify:

▪ Diarrhea

▪ Abdominal pain

▪ Gastric pain

▪ Constipation

▪ Other, specify ____________

If yes, specify the invasiveness of side effects:

(1 = few effects, 10 = many side effects)

1 2 3 4 5 6 7 8 9 10

Have the side effects of the old therapy been reduced after switching to the new therapy?

▪ Yes

▪ No

▪ No side effects with either therapy

Has the number of tablets been reduced with the new therapy?

▪ Yes

▪ No

Is the reduction in tablets comfortable/important for the daily routine?

▪ Yes

▪ No

▪ Indifferent

Do you feel that diabetes is better controlled with the new therapy?

▪ Yes

▪ No

Are you satisfied with the new therapy overall?

▪ Yes

▪ No

▪ Indifferent

If yes, specify why:

▪ Reduction in the number of tablets

▪ Fewer tablets help me remember to take my medication

▪ Fewer side effects

▪ Better glycemic control: I think that my diabetes is better controlled

Would you recommend switching to metformin-ER?

▪ Yes

▪ No
